# Characteristics and Determinants of Partial Remission in Children with Type 1 Diabetes Using the Insulin-Dose-Adjusted A1C Definition

**DOI:** 10.1155/2014/851378

**Published:** 2014-08-31

**Authors:** Aurore Pecheur, Thierry Barrea, Valérie Vandooren, Véronique Beauloye, Annie Robert, Philippe A. Lysy

**Affiliations:** ^1^Pediatric Endocrinology Unit, Cliniques Universitaires Saint Luc, Université Catholique de Louvain, Avenue Hippocrate 10, 1200 Brussels, Belgium; ^2^Pôle Epidémiologie et Biostatistique, Institut de Recherche Expérimentale et Clinique, Université Catholique de Louvain, Avenue Hippocrate 10, 1200 Brussels, Belgium

## Abstract

To evaluate the characteristics and determinants of partial remission (PR) in Belgian children with type 1 diabetes (T1D), we analyzed records of 242 children from our center. Clinical and biological features were collected at diagnosis and during follow-up. PR was defined using the insulin-dose-adjusted A1C definition. PR occurred in 56.2% of patients and lasted 9.2 months (0.5 to 56.6). 25.6% of patients entered T1D with DKA, which correlated with lower PR incidence (17.6% versus 82.3% when no DKA). In our population, lower A1C levels at diagnosis were associated with higher PR incidence and in young children (0–4 years) initial A1C levels negatively correlated with longer PR. Early A1C levels were predictive of PR duration since 34% of patients had long PRs (>1 year) when A1C levels were ≤6% after 3 months whereas incidence of long PR decreased with higher A1Cs. C-peptide levels were higher in patients entering PR and remained higher until 3 years after diagnosis. Initial antibody titers did not influence PR except for anti-IA2 titers that correlated with A1C levels after 2 years. Presence of 2 versus 1 anti-islet antibodies correlated with shorter PR. PR duration did not influence occurrence of severe hypoglycemia or diabetes-related complications but was associated with lower A1C levels after 18 months. We show that, at diagnosis of T1D, parameters associated with *β*-cell mass reserve (A1C, C-peptide, and DKA) correlate with the occurrence of PR, which affects post-PR A1C levels. Further research is needed to determine the long-term significance of PR.

## 1. Introduction

Type 1 diabetes is characterized by progressive autoimmune destruction of pancreatic *β* cells [1] that leads to symptoms of insulinopenia when *β*-cell mass is deficient. Glucose homeostasis is restored with administration of exogenous insulin and some patients experience a reduction of their daily insulin requirements (DIR) with maintenance of optimal glycemia and HbA_1C_ (A1C) levels [2]. Due to its impact on disease control, this phenomenon is called “partial remission” (PR) or “honeymoon period” and is variable both in intensity (with sometimes interruption of insulin) and duration [3]. Definition of PR was recently revised based on DIR and A1C levels (i.e., insulin-dose-adjusted A_1C_, IDAA1C) [4], after evidence of strong correlation between IDAA1C and stimulated C-peptide levels 6 and 12 months after diagnosis. Underlying mechanisms of PR remain largely unknown, but restoration of residual *β*-cell mass activity [5] in an otherwise insulin-sensitive environment [3] is believed to account for the condition. PR has driven much interest as a crucial target period for interventions aiming at preserving the residual *β*-cell mass, but immunomodulation trials have not proven to be efficient so far except for secondary study endpoints [6].

Besides potential therapeutic implications, the significance of PR is unclear, in terms of both disease progression and complication risks. Reduction of diabetes-related complications (retinopathy, nephropathy) has been described in patients with residual C-peptide secretion [7], but association factors are unknown and regular C-peptide measurements are included neither in current PR definition nor in clinical routine guidelines [8]. Moreover, PR impacts preliminary experiences of patients with type 1 diabetes, which change abruptly (with rise of both DIR and A1C) as PR ends. The objectives of this retrospective study are to evaluate the characteristics and potential determinants of PR according to the new definition [4] and to evaluate the presence of correlations between PR and long-term type 1 diabetes evolution (metabolic control and complications) and occurrence of severe hypoglycemia.

## 2. Patients and Methods

In our study, we included a total of 242 children aged 0.9 to 16.4  years, followed up in our pediatric diabetes clinic from diagnosis (established from 1994 to 2008) to adulthood (18–20  years of age). Type 1 diabetes was diagnosed according to International Society for Pediatric and Adolescent Diabetes guidelines and based on symptoms of insulinopenia, elevated blood glucose (BG) and A1C, positive anti-islet antibodies (GAD65, IA2, and insulin), and lack of family history of genetic diabetes. Biometrics (age, height *z*-score, and BMI *z*-score) and biological features (BG, A1C) were collected at diagnosis and at each consultation (postdiagnosis consultations occurred at 15 days and 1 month and then every 3 months; only fully adherent patients were recorded). At diagnosis, measures included screening of DKA (defined as pH < 7.3 and/or bicarbonate < 16 mM) and postprandial C-peptide levels (AutoDELFIA C-peptide, PerkinElmer Life and Analytical Sciences), which were assayed every year. *Z*-scores for height and BMI were assessed using Belgian Flemish reference charts. A1C was determined by high-capacity liquid chromatography with iron-resin exchange. BMI was calculated by using the formula (BMI = weight [kg]/height [m]^2^).

PR was defined as IDAA1C ≤ 9, according to definition by Mortensen et al. [4]: A1C (%) + [4 × insulin dose (U/kg/day)]. Insulin doses were adjusted for pre- and postprandial glycemic targets according to ISPAD guidelines, when available, or to our institution's guidelines. Screening for complications was performed yearly, starting from 2 years after diagnosis until adulthood, and included determination of microalbuminuria (urine spot), ophthalmoscopy, peripheral and autonomic neuropathy by physical examination, and blood lipids. Blood pressure was measured at each consultation. A patient was considered having complications when a single anomaly or more anomalies were diagnosed. Severe hypoglycemia was defined as loss of consciousness, coma with or without convulsions, or alteration of consciousness impeding the capacity for oral sugar ingestion (need of a tier for IM glucagon administration). Occurrence of severe hypoglycemia was monitored at each consultation (as per our institution's guidelines).

For data analysis, 3 age subgroups were constituted (0–4  years, 5–9  years, and >10  years) in order to include most pubertal patients into one subgroup (>10  years).

Data were analyzed using the GraphPad software. Categorical variables were analyzed using chi-square test and continuous variables were analyzed using chi-square trend test, unpaired *t*-test, or Mann-Whitney *U* test, according to the statistical distribution. Data were submitted to D'Agostino and Pearson omnibus normality test and Levene's test for equality of variances. ANOVA with *R* tests were used when there were more than two groups. Multiple comparisons were subsequently conducted when significant. Changes over time were compared using Student's paired *t*-test. Correlation analysis was used to evaluate relationship between variables. To assess the relative contribution of each variable (initial C-peptide, initial A1C, sex, and DKA at diagnosis) to chances of remission, logistic regression models were built. All significant variables in univariate analyses were entered into a multivariate logistic regression. Results are expressed as odds ratio (OR) with 95% confidence intervals. Logistic regression analyses were performed using IBM SPSS Statistics 21.0 software. *P* < 0.05 was considered significant. This study was approved by the local ethical committee.

## 3. Results

In our clinical series, PR occurred in 56.2% of patients with type 1 diabetes (Table 1), without any case of complete remission. The proportion of girls (47.5%) and boys (52.5%) was comparable among remitters and nonremitters. Mean age at diagnosis was 8.8 ± 3.8 years with no difference between subgroups (PR versus no PR, girls, boys, and age subgroups). Height and BMI *z*-scores, measured at first follow-up consultation to avoid influence of insulinopenia on weight, were also similar between subgroups and to the distribution of healthy Belgian children from the reference database.

At diagnosis, most patients had no DKA (74.4% versus 25.6% with DKA, *P* < 0.0001) and the absence of DKA was significantly higher in PR (82.3%) than in no PR (63.5%) patients (*P* = 0.0047) (Table 2). This effect was more pronounced in boys, who had a higher PR frequency (69.4% versus 30.6% no PR, *P* = 0.0023) when no DKA occurred at diagnosis. Furthermore, multivariate logistic regression analysis showed that chances of PR were higher when there was no DKA at diagnosis (OR = 0.43, *P* = 0.018) (cf. below and in Table 3).

**Figure 1:**
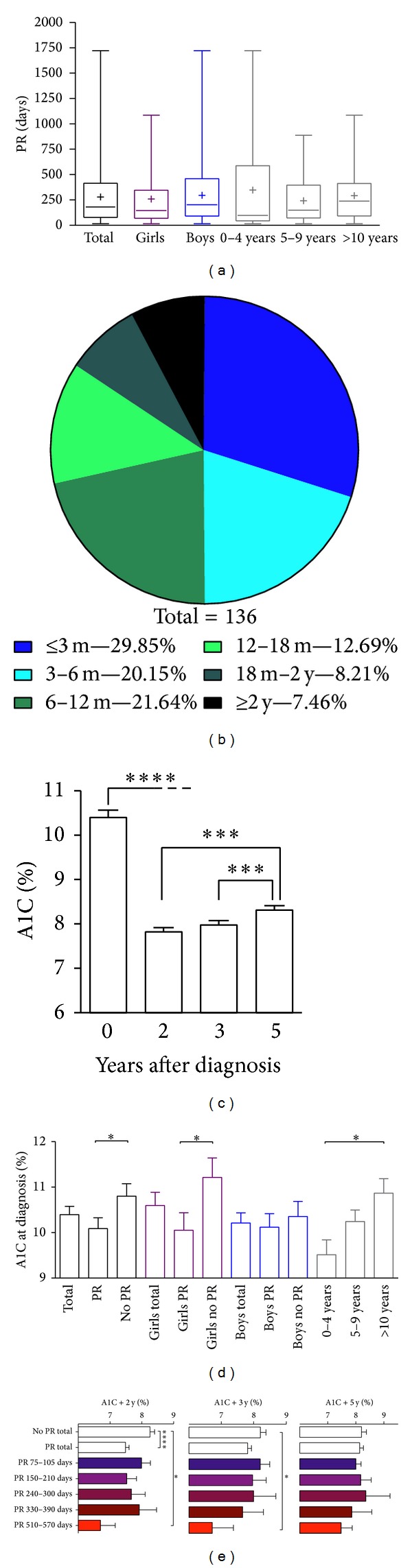
Characteristics of PR, A1C at diagnosis and during follow-up. (a) Box plots with ranges showing no difference in PR duration between gender and age subgroups. [+] shows means for each group. (b) Distribution of PR durations among remitters (*n* = 136). (c) Evolution of A1C levels during follow-up. (d) A1C levels at diagnosis among gender and age subgroups. (e) Graphs showing correlation between PR duration and A1C levels 2, 3, and 5 years after follow-up (resp., A1C+2y, A1C+3y, and A1C+5y). PR durations were grouped to correspond to 3 months ± 15 days and 6, 9, 12, and 18 months ± 30 days. All bars were shown with SEM. *P < 0.05, ***P < 0.001, and ****P < 0.0001 compared with indicated groups.

Mean PR duration was 279.6 days (range 15–1722) or 9.2 months (range 0.5–56.6) with no differences between gender and age subgroups (Figure 1(a)). In 71.6% of patients, PR duration was shorter than 1 year and PR mostly occurred during the first 6 months (Figure 1(b)). However, 7.5% of patients experienced PR during more than 2 years (mean 992 days; 95% CI [797, 1187]).

At diagnosis, patients had an overall A1C at 10.4 ± 2.8% (Figure 1(c)), which then dropped after 2, 3, and 5 years. Patients entering PR had a lower baseline A1C (10.1 ± 2.7%) than patients with no subsequent PR (10.8 ± 2.8%) (*P* = 0.049) (Figure 1(d)). This effect was evidenced in girls entering PR who had a lower A1C at diagnosis (10.0 ± 2.9%) than girls without PR (11.2 ± 3.1%) (*P* = 0.028). However, boys had similar A1C levels whether they entered PR or not. In multivariate logistic regression models, lower A1C at diagnosis was associated with higher chances of PR (OR = 0.87, *P* = 0.03) (Table 3), but levels of A1C at diagnosis did not correlate with PR duration when analyzed for all patients (cf. below for linear regression analyses). Yet when age subgroup 0–4  years was considered, A1C levels at diagnosis negatively correlated with longer PR durations (*P* = 0.01, *r* ^2^ = 0.15). Initial A1C did not impact long-term A1C levels since no correlation could be observed between A1C at diagnosis and A1C at 2 years after diagnosis (A1C+2y) or at 3 years after diagnosis (A1C+3y), either in PR or in no PR subgroups (see Figures S1(a)-S1(b) in Supplementary Material available online at http://dx.doi.org/10.1155/2014/851378).

A1C+2y was significantly lower in the PR (7.5%; 95% CI [7.3, 7.7]) than in the no PR population (8.2%; 95% CI [7.9, 8.5]) (*P* < 0.0001) (Figure 1(e)), which is partly explained by the fact that PR duration is longer than 2 years in 7.5% of the PR patients. This observation was corroborated by the fact that A1C at 3 and 5 years after diagnosis (resp., A1C+3y and A1C+5y) did not differ between PR and non-PR groups. However, A1C+3y was lower (6.7%; 95% CI [4.7, 8.8]) in patients having a remission lasting between 510 and 570 days as compared to nonremitters (8.2%; 95% CI [7.8, 8.5]) (*P* = 0.045), suggesting a prolonged effect on A1C after the PR period in this subgroup.

**Figure 2:**
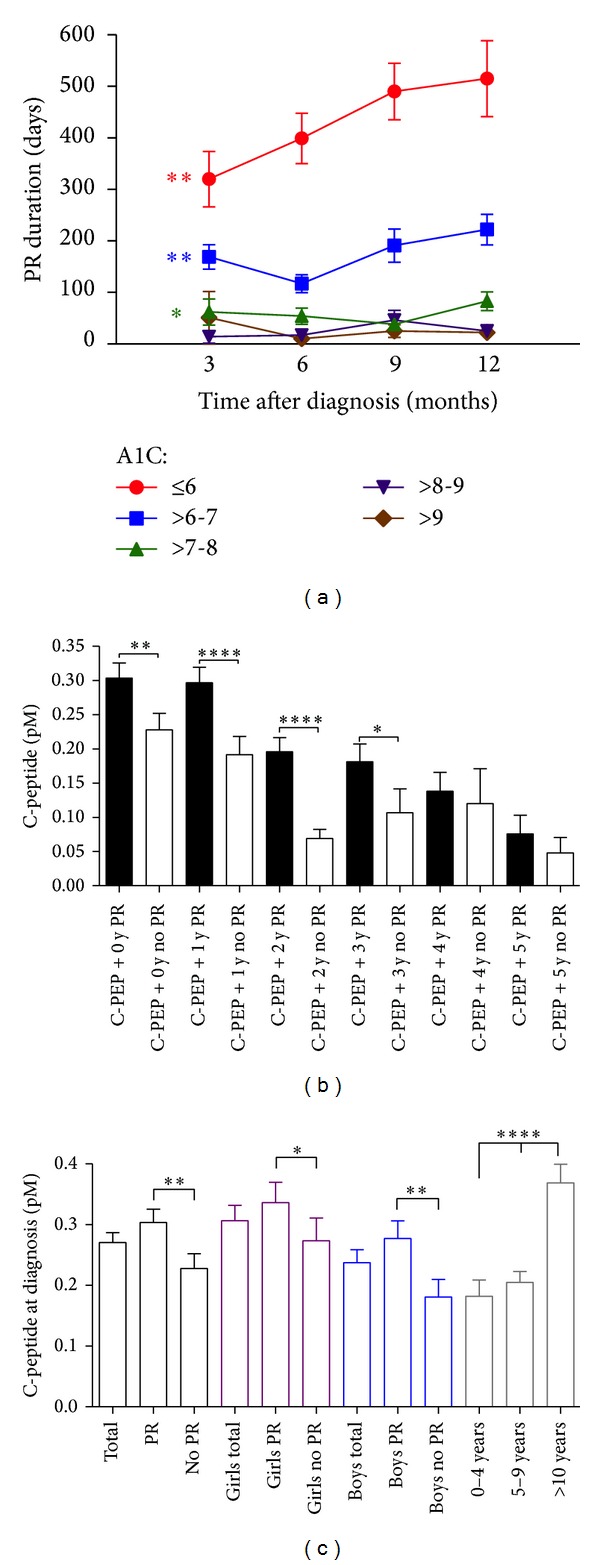
Influence of early A1C levels on PR duration and characteristics of C-peptide levels among PR, age, and gender subgroups. (a) Graph showing negative correlation between A1C levels (subgroups indicated) at 3-, 6-, 9-, and 12-month follow-up and PR duration. (b) C-peptide levels were higher in patients entering PR when evaluated at diagnosis and after 1, 2, and 3 years. (c) Differences in C-peptide levels among PR, gender, and age subgroups. All bars were shown with SEM. *P < 0.05, **P < 0.01, and ****P < 0.0001 compared with indicated groups.

Furthermore, A1C levels at 3, 6, 9, and 12 months after diagnosis were strongly associated with PR duration when subgrouped (Figure 2(a)). Indeed, low (≤6%) A1Cs at 3 to 12 months were associated with longer PR (mean 320 to 515 days), whereas PR durations dropped to mean 169 to 222 days for A1Cs > 6 to 7% (*P* < 0.01) and to mean 62 to 82 days for A1Cs > 7 to 8% (*P* < 0.01). With high A1Cs (>8 to 9%), PR durations were only negligible (14–46 days, *P* < 0.05) and not different from patients with poorly controlled diabetes (A1C > 9%). Hence, A1C levels at 3 months allowed us to estimate the proportion of patients that will experience PR for more than 1 year at 34% (*n* = 15/44) when A1C levels were ≤6%, 16% (*n* = 16/102) for A1Cs > 6-7%, and 5% (*n* = 2/36) for A1Cs > 7-8%. Similarly, at 6-month follow-up, these proportions changed to 46% (*n* = 23/50) for A1Cs ≤ 6%, 8% (*n* = 6/74) for A1Cs > 6-7%, and 4% (*n* = 2/50) for A1Cs > 7-8%.

No differences could be observed in the titers of anti-GAD65 and anti-IA2 antibodies at diagnosis among the different analyzed populations (girls, boys, and age subgroups) (Table S1). However, PR duration was significantly shorter (85.8 days; 95% CI [33.9, 137.6]) in patients having 2 positive anti-GAD65 and anti-IA2 antibodies at diagnosis compared with patients with only 1 positive antibody (198.0 days; 95% CI [70.9, 325.0]) (*P* = 0.04). The effects of 2 antibodies versus 1 remained when accounting for DKA. Additionally, anti-IA2 antibody titers positively correlated with A1C+2y levels (*P* = 0.006, *r* ^2^ = 0.16), but this correlation was not found with A1C+3y and A1C+5y levels or with the other antibodies, partly due to smaller sample sizes.

C-peptide secretion was significantly different in PR versus no PR patients, from diagnosis until 3 years after diagnosis (Figure 2(b)). However, this difference could not be observed after 4 and 5 years after diagnosis, perhaps in part because of a bias of patient selection for C-peptide screening. Higher baseline C-peptide concentrations were observed both in girls and in boys entering PR compared to patients without PR and this effect was more pronounced in boys (Figure 2(c)). An age effect on C-peptide levels was observed not only at diagnosis where higher C-peptide levels were found in patients aged >10  years, as compared with other age groups (0–4  years, 5–9  years) (*P* < 0.0001) (Figure 2(c)), but also later on since age at diagnosis positively correlated with C-peptide levels at 2 years after diagnosis (*P* < 0.0001, *r* ^2^ = 0.21).

In multivariate logistic regression, other variables than A1C levels and DKA at diagnosis (i.e., age, sex, height and BMI *z*-scores, C-peptide levels, and anti-GAD65 and IA2 titers) were not significantly associated with a higher chance of PR (Table 3). When PR was considered on a continuous scale (PR duration), none of the estimates was significant in linear regression, even if consistent with logistic regression estimates.

PR duration or A1C+2y levels did not correlate with occurrence of severe hypoglycemia as these parameters were not different in patients without documented episodes of severe hypoglycemia during the follow-up period as compared to patients having at least one episode (*n* = 97; 39.7%) or more than 2 (*n* = 32; 13.2%) episodes of severe hypoglycemia. Furthermore, only few patients (*n* = 11) developed diabetes-related complications during follow-up until adulthood; thus, no differences with complication-free patients could be observed in terms of PR duration and  A1C+2y.

## 4. Discussion

In our study, overall occurrence of PR was 56.2% and mean PR duration was 9.2 months, which was comparable to other studies using IDAA1C or DIR and A1C levels as PR definition (Table S2). Table S2 shows studies that included A1C and DIR in PR definition since PR defined with DIR only [9] or A1C only [7] overestimates PR rates.

While some data [10] showed that young children (<5  years) have lower rates of PR, in our study, young children (0–4  years) had similar PR rates (47.7%) than older children (60.2% and 56.3%, resp., in children aged 5–9  years and >10  years). Similar to other studies [10, 11], even with different PR definitions [7, 12], we did not find gender differences. However, Dost et al. reported longer PR in boys below 10  years of age [13], but in the same study PR durations were shorter for patients <10  years when the global cohort was analyzed. Furthermore, we did not observe any complete remission as per IDAA1C definition or defined as a complete interruption of insulin administration, contrary to data from other groups [11, 14] showing complete remission in up to 4% of patients for a mean period of 3 months [11].

Our finding that DKA is associated with a lower frequency of PR is in concordance with the literature. However, this effect was more pronounced in boys than in girls in our series. Furthermore, we did not find a higher frequency of DKA in younger children (0–4  years) as described elsewhere [9, 10, 15, 16]. Whether DKA is a variable independent of PR is unclear, since DKA might reflect rapid *β*-cell destruction [17] and thus preclude ongoing PR development, by contrast to patients without DKA whose *β*-cell mass is partially preserved.

In our study, higher initial A1C levels were associated with higher chances of PR. However, only girls had lower A1C levels at diagnosis when entering PR, and only young patients (0–4  years) had their A1C levels at diagnosis correlating negatively with PR duration. We did not find similar gender- or age-related correlations in the literature, whether as it was not measured or not found [11]. Interestingly, our findings show that early A1C levels might be helpful to predict PR durations since, as soon as 3 months after diagnosis, lower A1Cs were significantly associated with a higher incidence of longer PRs, this association being present at 6-, 9-, and 12-month follow-up.

Likewise to A1C, patients entering PR had higher C-peptide levels at diagnosis, and this effect was more pronounced in older (>10  years) patients. Also, older children at diagnosis had more chance to have higher C-peptide levels after 2 years. However, initial C-peptide levels were not significantly associated with higher chances of PR. In our study, we randomly assayed postprandial C-peptide levels, making definitive analysis of *β*-cell reserve difficult. Proper analysis of stimulated C-peptide with mixed meal tolerance testing was not available in our retrospective study. However, since C-peptide secretion is not affected by exogenous insulin and higher C-peptide levels may be reached by random rather than stimulated sampling [18], we believe that our data provide a valuable basis for correlation analyses. Furthermore, the age effect in C-peptide levels observed in our series might in part be explained by differences in treatment modalities. Indeed, in our center during the study period, adolescent patients were systematically proposed multiple daily injections (MDI) whereas younger children were offered* bis in die* insulin regimens. Whereas similar glycemic and A1C targets were applied in both groups, MDI-treated patients might have benefited from intensive insulin therapy which was shown to prolong C-peptide levels in the DCCT trial [19]. Nevertheless, as for DKA, A1C and C-peptide levels at diagnosis may perhaps not be considered independent of PR, since they reflect, directly or not, residual *β*-cell mass.

While young children were shown to have either higher [16] or lower [15] rates of anti-islet antibodies at diagnosis of type 1 diabetes, we found no difference in anti-GAD65 or anti-IA2 titers in any of the analyzed subgroups. Only in patients without PR and aged 0–4  years a trend towards higher anti-GAD65 titers was observed, while this was not significant (*P* = 0.06). Our finding that PR duration was shorter in patients with 2 positive autoantibodies, as compared to patients with a single autoantibody, corroborated a recent study [20] showing higher probability for developing type 1 diabetes in children having 2 anti-islet antibodies (69.7%) versus one autoantibody (14.5%). In this prospective cohort, progression towards type 1 diabetes after seroconversion was also accelerated in the group having 2 autoantibodies whereas no data were provided regarding evolution of type 1 diabetes after diagnosis.

Finally, we did not observe long-term effects of PR on diabetes complications or incidence of severe hypoglycemia, partly due to low numbers of events. Yet a short-term effect of PR on metabolic control was suggested in patients having PR durations between 510 and 570 days, since they had lower A1C levels after 3 years. However, no difference in A1C levels was detected after 5 years, showing the rapid loss of this effect.

In conclusion, our comprehensive analysis of clinical and biological features of newly diagnosed Belgian type 1 diabetes patients shows that, at diagnosis, parameters directly or indirectly associated with *β*-cell mass reserve (A1C and DKA) correlate with PR and that longer PR duration is associated with a lower A1C even 18 months after PR ends. Further long-term research is needed to determine if this effect is associated with a reduced rate of complications and severe hypoglycemia.

## Supplementary Material

Graphs showing the absence of correlation between A1C at diagnosis and A1C at 2 (A) or 3 (B) yr after diagnosis, when compared for non-remitters or for patients that experienced PR for <1 yr, 1-2yr, and >2 yr.

## Figures and Tables

**Table 1:** Characteristics of the clinical series at diagnosis.

	Total (*n* = 242)	PR (*n* = 136)	No PR (*n* = 106)	*P* ^ a^
Sex—*n* (%)				0.35
Girls	115 (47.5)	61 (44.8)	54 (50.9)	
Boys	127 (52.5)	75 (55.1)	52 (49.1)	
Age at Δ				
Mean—year^b^	8.8 ± 3.8	8.9 ± 3.8	8.9 ± 3.9	0.96
Median—year	9.5	9.4	9.6	
Range—year	0.9–16.4	1.4–16.4	0.9–16.4	
Girls—year^b^	9.4 ± 3.4	9.3 ± 3.5	9.5 ± 3.4	0.75
Boys—year^b^	8.5 ± 4.2	8.6 ± 4.1	8.2 ± 4.5	0.65
0–4 years—*n* (%)	44 (18.2)	21 (15.4)	23 (21.7)	0.50
5–9 years—*n* (%)	93 (38.4)	56 (41.2)	37 (34.9)	
>10 years—*n* (%)	103 (42.6)	58 (42.6)	45 (42.4)	
Height *z*-score at Δ				
Girls	0.0 ± 1.2	0.0 ± 1.2	0.0 ± 1.1	0.72
Boys	0.0 ± 1.2	0.0 ± 1.0	−0.2 ± 1.4	0.36
BMI *z*-score at Δ				
Girls	0.0 ± 1.1	−0.1 ± 0.9	+0.1 ± 1.2	0.34
Boys	+0.3 ± 1.1	+0.3 ± 1.0	+0.3 ± 1.3	0.64

^a^Categorical variables were analyzed using chi-square test; continuous variables were analyzed using chi-square test with trend; ages at diagnosis were analyzed using unpaired *t*-test. ^b^Plus-minus values are means ± SD. Δ: diagnosis.

**Table 2:** Subgroup analysis of DKA occurrence.

		PR (*n* = 102)	No PR (*n* = 74)	*P* value^a^
Total (*n* = 176)				
DKA—*n* (%)	45 (25.6)	18 (17.6)	27 (36.5)	0.0047
0–4 years	11 (24.4)	3 (16.7)	8 (29.6)	0.69
5–9 years	18 (40)	9 (50)	9 (33.3)	
>10 years	16 (35.6)	6 (33.3)	10 (37)	
Girls (*n* = 88)				
DKA—*n* (%)	19 (21.6)	7 (6.9)	12 (16.2)	0.08
No DKA—*n* (%)	69 (78.4)	41 (40.2)	15 (37.8)	
Boys (*n* = 88)				
DKA—*n* (%)	26 (29.5)	11 (10.8)	15 (20.3)	0.017
No DKA—*n* (%)	62 (70.5)	43 (42.1)	19 (25.7)	

^a^Compared occurrence of DKA and non-DKA among subgroups (total, girls, boys, and age subgroups). Categorical variables were analyzed using chi-square test; continuous variables were analyzed using chi-square test with trend.

**Table 3:** Factors at diagnosis associated with PR, using multivariate logistic regression.

	Univariate unadjusted			Multivariate adjusted		
	OR	95% CI	*P* value	OR	95% CI	*P* value
Age—year	1.03	[0.96; 1.11]	0.43			
Boys (yes: 1, no: 0)	1.39	[0.76; 2.52]	0.29			
Height *z*-score	1.17	[0.84; 1.63]	0.36			
BMI *z*-score	0.94	[0.71; 1.23]	0.64			
C-peptide (pM)	2.20	[0.60; 8.05]	0.24			
A1C (%)	0.86	[0.76; 0.97]	0.014	0.87	[0.77; 0.99]	0.03
DKA (yes: 1, no: 0)	0.39	[0.19; 0.77]	0.007	0.43	[0.21; 0.86]	0.018
*α*GAD65—IU/mL	0.99	[0.98; 1.01]	0.46			
*α*IA2—IU/mL	0.98	[0.94; 1.02]	0.22			

CI: confidence interval; OR: odds ratio; Δ: diagnosis.
