# Supplementary material for: Characteristics and Determinants of Partial Remission in Children with Type 1 Diabetes Using the Insulin-Dose-Adjusted A1C Definition
Source: J Diabetes Res. 2014 Aug 31;2014:851378. doi: 10.1155/2014/851378 (PMC4164125; doi:10.1155/2014/851378)
Supplement: Supplementary file 1 — Graphs showing the absence of correlation between A1C at diagnosis and A1C at 2 (A) or 3 (B) yr after diagnosis, when compared for non-remitters or for patients that experienced PR for <1 yr, 1-2yr, and >2 yr. [file 851378.f1.pdf]

Supporting Information

Characteristics And Determinants Of Partial Remission In Children With Type 1 Diabetes using the Insulin-Dose-Adjusted A1C Definition

Aurore Pecheur, Thierry Barrea, Valérie Vandooren, Véronique Beauloye, Annie Robert, Philippe A. Lysy

Figure S1

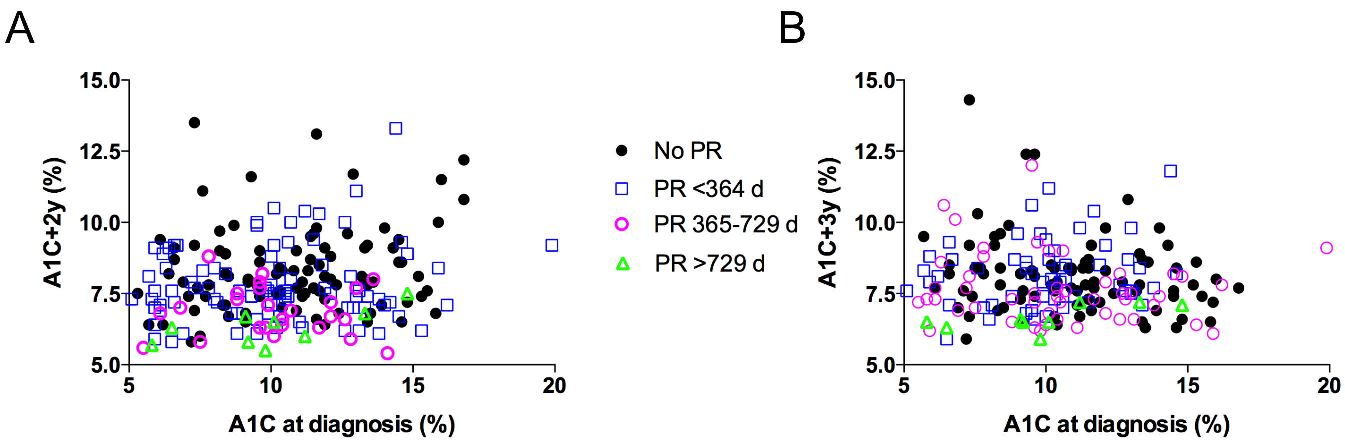
A
A1C+2y (%)
15.0
12.5
10.0
7.5
5.0
5
10
15
20
A1C at diagnosis (%)
No PR
PR <364 d
PR 365-729 d
PR >729 d
B
A1C+3y (%)
15.0
12.5
10.0
7.5
5.0
5
10
15
20
A1C at diagnosis (%)

Figure S1: Influence of A1C levels at diagnosis on PR duration and A1C at 2 years follow-up

Graphs showing no correlation between A1C levels at diagnosis, PR duration subgroups and A1C+2y (**A**) or A1C+3y (**B**).

Table S1

	Total (IU/mL)	PR (IU/mL)	No PR (IU/mL)	*P*
αGAD65 Abs	4.5 (0.0 – 275.0)	4.7 (0.0 – 275.0)	4.5 (0.0 – 214.8)	0.99
Girls	4.9 (0. – 96.3)	4.8 (0.0 – 71.6)	5.1 (0.0 – 96.3)	0.74
Boys	2.3 (0.0 – 275.0)	2.7 (0.0 – 275.0)	2.3 (0.0 – 214.8)	0.78
0-4 yr	2.5 (0.0 – 214.8)	0.68 (0.0 – 9.3)	5.6 (0.0 – 214.8)	0.06
5-9 yr	4.2 (0.0 – 149.0)	4.4 (0.0 – 55.5)	2.3 (0.0 – 149.0)	0.98
>10 yr	4.9 (0.0 – 275.0)	5.5 (0.0 – 275.0)	4.5 (0.0 – 96.3)	0.41

	Total (IU/mL)	PR (IU/mL)	No PR (IU/mL)	*P*
αIA2 Abs	4.5 (0.0 – 93.0)	2.7 (0.0 – 93.0)	5.3 (0.0 – 71.7)	0.31
Girls	2.4 (0.0 – 48.9)	2.3 (0.0 – 48.9)	3.2 (0.0 – 28.7)	0.84
Boys	5.2 (0.0 – 93.0)	3.1 (0.0 – 93.0)	15.7 (0.0 – 71.7)	0.18
0-4 yr	4.5 (0.0 – 71.7)	4.5 (0.0 – 22.6)	22.6 (0.3 – 71.7)	0.62
5-9 yr	5.3 (0.0 – 93.0)	2.4 (0.0 – 93.0)	9.3 (0.6 – 24.3)	0.47
>10 yr	9.6 (0.0 – 44.2)	1.8 (0.0 – 44.2)	4.5 (0.0 – 28.7)	0.79

Data are expressed as median with range. Antibody titers were analyzed using Mann-Whitney test. α: anti-; abs: antibodies.

Table S2

	Abdul-Rasoul 2006	Dost 2007	Bowden 2008	Mortensen 2009	Neylon 2013
Study center(s)	Kuwait	Germany	Ohio, USA	15 countries	Australia
Study design	Prospective	Retrospective	Retrospective	Prospective	Retrospective
Enrollment dates	4/2000 – 5/2003		1/2004 – 12/2004	8/1999 – 12/2000	1/2005 – 1/2006
n	103	6123	152	275	109
Age – yr	6.6 ± 3.9 (<12)	<18	3 subgroups (< 5, 5-12, >12)	9.2 ± 3.7 (<16)	9.0 ± 3.4
Female	62.1%		33.3 to 50%	ND	45%
PR characteristics					
Definition	DIR <0.5 U/kg + A1C ≤6%	DIR <0.5 U/kg + A1C ≤7%	DIR <0.5 U/kg + A1C <8%	IDAA1C	IDAA1C
Occurrence	68.9%	32.5%	<5 yr: 26.8%;<br>5-12 yr: 56.6%;<br>>12 yr: 29.4%	<4.9 yr: ≈45%;<br>5-9.9 yr: ≈60%;<br>≥10 yr: ≈70%	35%
Duration	7.2 months (95% CI, 2.3-12.3)	0.74 ± 0.77 yr	ND (12 month follow-up)	ND (12 month follow-up)	8 months (range 3-26)
Independent PR determinants	(+) age 5-12 yr<br>(−) DKA (no DKA = 100% PR)<br>(−) duration of symptoms before Δ<br>(−) BG, acidosis at Δ	(+) Boys (< 10 yr)<br>(+) Puberty<br>(−) age <10 yr	(+) No DKA at Δ<br>(−) age <5 yr and >12 yr	(+) stimulated C-peptide at 6 and 12 months<br>(−) age <4.9 yr	(+) age (*r* = 0.26)
Remarks	- no gender differences<br>- no PR in patients <3 yr<br>- 4% total remission<br>- DKA 84.5%		- DKA 27.4 to 46.3%<br>- No correlation between gender or BG, Abs and A1C at Δ and PR rate		- no PR in patients <3.5 yr<br>- more DKA in patients <3.5 yr<br>- DKA 33%

Abs: antibodies; BG: blood glucose; Δ: diagnosis; DIR: daily insulin requirements; ND: not determined.
